# Malnutrition in rural Solomon Islands: An analysis of the problem and its drivers

**DOI:** 10.1111/mcn.12921

**Published:** 2020-01-31

**Authors:** Joelle Albert, Jessica Bogard, Faye Siota, Joe McCarter, Salome Diatalau, Josephine Maelaua, Tom Brewer, Neil Andrew

**Affiliations:** ^1^ WorldFish Honiara Solomon Islands; ^2^ Agriculture and Food, Commonwealth Scientific and Industrial Research Organisation (CSIRO) St Lucia Queensland Australia; ^3^ Melanesia Program, Wildlife Conservation Society Fiji; ^4^ Center for Biodiversity and Conservation, American Museum of Natural History United States of America; ^5^ Solomon Island Government, Ministry of Health and Medical Services Honiara Solomon Islands; ^6^ Solomon Islands National University Honiara Solomon Islands; ^7^ Australian National Centre for Ocean Resources and Security University of Wollongong Australia

**Keywords:** malnutrition, nutritional status, dietary diversity, Pacific Islands, food system, participatory action research, nutrition sensitive agriculture

## Abstract

Solomon Islands, like many Pacific Island nations, suffer from the burden of malnutrition. External drivers including population growth, declining agriculture and fisheries productivity and global food trade have contributed to the transition to greater reliance on imported foods. Globally, diets are recognized as both a cause of and solution to the burden of malnutrition. Using a mixed‐method approach this study assessed nutritional status and key determinants of malnutrition among women and young children in rural Solomon Island communities. Quantitative 24‐hour recall surveys identified diets of women and young children in these communities to be very limited in diversity. Typical daily diets comprised of fish, sweet potato (and/or rice) and slippery cabbage (a leafy green) usually boiled in coconut milk or baked. Participatory research using problem tree and biocultural approaches identified basic determinants of poor diets and opportunities to address these challenges. We highlight three domains of opportunity to improve diets across multiple scales; 1) improve nutrition‐sensitive agriculture and fisheries to produce and distribute diverse, productive and nutrient rich foods; 2) nutrition education and empowerment, focusing on the first 1000 days of life, to influence and inform choices regarding food consumption; and 3) reducing the consumption of imported, energy‐rich nutrient poor foods through national and regional policies. These multi‐scale domains highlight that food system approaches that strengthen integrated policy and empower people are essential for healthy and sustainable diets in Solomon Islands and more broadly in the Pacific region.

**Key messages**

- The triple burden of malnutrition impacts the lives and development opportunities of many people in the Pacific region.
- Dietary diversity of women and young children in rural Solomon Islands is poor, with diets likely to be inadequate in key micronutrients.
- Participatory research with communities identified basic determinants of poor‐quality diets and opportunities to address these challenges.
- Food systems approaches that strengthen integrated policy and community action for healthy and sustainable diets are essential to address nutrition security in Solomon Islands and the Pacific region.

## 1 INTRODUCTION

Malnutrition afflicts one in three people globally, and is among the leading causes of the global burden of disease (Gakidou et al., 2017; Development Initiatives, 2018; HLPE 2017). Maternal and child malnutrition is acute in developing countries and has profound consequences for people's lives and the societies and economies in which they live (Victora et al. 2008; Black et al., 2013). South East Asia and the Pacific region are home to nearly half the world's population experiencing the double burden of malnutrition (Haddad, Cameron, & Barnett, 2015). Food and nutrition security are prominent in the development aspirations articulated in the Sustainable Development Goals and through them the world leaders have committed to ending all forms of malnutrition (United Nations, 2015; WHO, 2017).

Solomon Islands is an archipelago of more than 1000 islands in the south‐west Pacific Ocean. The country is ranked 152^nd^ on the human development index and is among the least developed countries in the world (UNDP, 2018). Most Solomon Islanders live in rural, coastal communities where fish is the primary animal‐source food and gardens provide root crops and vegetables for household needs and income generation (Andersen et al., 2013; Bourke et al., 2006; Govan, Schwarz, Harohau, & Oeta, 2013; McCarter et al., 2018). Despite this largely subsistence mode of living, rapid population growth, shortages of arable land, declining fish stocks and cheap, low‐quality food imports create challenges for nutrition security (Horsey, et al., 2019; Hughes & Lawrence, 2005; C‐POND, SPC, UNDP, & WHO, 2013; Snowdon & Thow, 2013; Charlton et al., 2016).

Malnutrition in Solomon Islands manifests as stunting in children under 5 years of age, adult obesity, and iron deficiency anemia in women of reproductive age (referred to as women) and young children (SINSO, MHMS, & SPC, 2017). Diets that are inadequate in vitamins and minerals, and frequent exposure to infections contribute to the prevalence of child stunting (Andersen, Thilsted, & Schwarz, 2013).

Globally, diet is recognized as both a cause of, and solution to, the global burden of malnutrition (Development Initiatives, 2018). Despite the central position of nutrition security in international, regional and national development ambitions, there remain large gaps in the empirical foundation needed to guide policy and more local interventions in the food environment. This study contributes to filling this gap by describing dietary patterns and exploring underlying perceptions of nutritional issues and their determinants in rural Solomon Island communities. We focus our mixed‐methods analysis on women and children who are often the most nutritionally vulnerable population groups.

## 2 METHODS

### 2.1 Description of study area

This study was undertaken in four rural communities in Malaita and Western Province, Solomon Islands. Despite differences in population, language, religion and culture, land and sea are the common livelihood platform across the four communities. Malaita is the most populated province in the country, and has experienced a rapid decline in soil fertility related to pressures of population growth, reduced fallow periods (Kabu, 2001; Allen et al., 2006) and declining productivity of coastal resources (Schwarz, Andrew, Govan, Harohau, & Oeta, 2013). In contrast, Western Province has fewer people, and has more ecologically diverse and relatively intact coastal fisheries. Commercial logging has almost exhausted the province's rich forest resources and logging royalties have resulted in a significant reliance on the cash economy (Allen et al., 2006; Bennett et al., 2014).

In Malaita Province, research focused on two remote communities in the Lau region of North Malaita. Lau people are traditionally recognized as *wane i asi* (saltwater people), but villages differ in their reliance on the sea. Community M1 was located on the mainland with good access to land for agriculture, albeit with declining soil fertility (Kabu, 2001). Households from M1 were within walking distance of Lau lagoon and its productive seagrass associated fishery. Community M2 was located on the island of Manobe, in the outer part of Lau lagoon. Manobe land is relatively unproductive and people are heavily reliant upon the sea ‐ selling or bartering fish with mainland people for root crops and vegetables.

The two communities in Western Province (W1 and W2) were situated on the exposed ocean‐facing side of Marovo Lagoon and were similar in some respects: both were relatively small (around 100 adults) and isolated (1‐2 hours by boat from a market town). Residents at both sites manage large ridge‐to‐reef conservation areas, and produce food from gardens close to their village. The main income sources are fresh garden produce and wage labour in logging operations. In W1, there is a significant eco‐tourism driven local handicraft market.

### 2.2 Approach

To understand nutrition issues and their basic determinants, we took a mixed methods approach guided by the UNICEF framework of malnutrition (UNICEF, 1990). This study therefore combines quantitative measures of nutritional status and its determinants based on surveys and anthropometric assessments; and community perspectives gathered using qualitative methods including focus group discussions (FGDs) and key informant interviews.

#### 2.2.1 Nutritional status and determinants

Quantitative surveys and anthropometric assessments were completed with eligible and consenting households in each community in May‐June 2016. An eligible household consisted of at least one women (aged 15 to 49 years) and/or young children under the age of 5 years. Interviews and FGDs were undertaken in pijin (the *lingua franca* of Solomon Islands) with translations to local language, as required.

During interviews household characteristics were enumerated, including number of adults and children, primary sources of income, education attainment of the household head and of women participating in the dietary diversity surveys, and ownership of household assets. Household data including housing construction materials, asset ownership (boats, mobile phones, radio, livestock), access to power (solar, generators), and access to water and sanitation facilities were used to calculate proxies for wealth using Principal Component Analysis (PCA; see section 2.3.2).

Indicators for nutritional status included the proportion of women overweight or obese and the prevalence of stunting, underweight and wasting in children under 5 years of age. Anthropometric measurements were taken from women, infants and young children (aged 6 to 23 months and 2 to 5 years). Body weight was measured using SECA 770 weighing scales to the nearest 0.01 kg and height using child/adult height boards to the nearest mm.

Based on WHO thresholds (WHO, 2010), adult body mass index (BMI) was used to determine the *proportion of* women *overweight or obese* (BMI > 25). Waist and hip circumference were used to calculate *waist to hip ratio*, with a ratio >0.85 for women indicating greater risk of developing serious health conditions. For infants and young children, following WHO (2006), age, weight and height were used to determine the prevalence of stunting, wasting and underweight.

Dietary quality of women was measured using the *minimum dietary diversity of women* (MDD‐W) indicator, a validated measure of micronutrient adequacy (Martin‐Prével et al., 2015). The MDD‐W indicator is calculated as the proportion of women that consumed five or more of ten key food groups (FAO & FHI 360, 2016). While a globally endorsed indicator of micronutrient adequacy, to the authors knowledge, this study is the first to measure MDD‐W in Solomon Islands (and broader Pacific region). As such the use of the ten food groups may provide a potential bias for understanding the micronutrient adequacy of Pacific Islander diets. For infants and young children aged 6 to 23 months, the *minimum dietary diversity for infants* (*IYCF MDD)* was used, defined as the proportion of infants and young children who consume foods from four or more of seven key food groups (WHO, 2008).

Household food consumption scores were calculated as an indicator of food availability and access. Food consumption scores were based on the frequency of consumption of key food groups (staples, pulses, vegetables, fruit, meat/fish, milk, sugar and oil) in the 7 days prior to interviews, using World Food Programme developed methodology (WFP, 2008). The quantities of rice, sugar and canned tuna consumed by households in the 7 days prior to interviews were also collected. We were confident that respondents accurately estimated quantities as in contrast to other common foods, these items are purchased either daily or weekly in standard units.

To assess child care resources and practices, we collected a number of standard WHO indicators for infant feeding practices including; *minimum meal frequency*, *minimal acceptable diet*, and whether a *child was ever breastfed* (WHO, 2008). Proxy indicators were used for adequate access to health resources (*time taken to reach the closest health clinic)* and water and sanitation facilities (*proportion of households interviewed with improved sanitation facilities* and *proportion of households with improved drinking water supply)*. For this study, improved sanitation facilities were defined as those that separated excreta from human contact and were not shared by more than two households. Common improved sanitation facilities are flush or pour septic systems and pit latrines with a concrete slab. Improved drinking water was defined as water sources protected from contamination, including rainwater tanks and public standpipes.

#### 2.2.2 Community perspectives of nutrition issues

We undertook qualitative participatory research to understand and document community perspectives on nutrition issues and their determinants. In Malaita we used a problem tree analysis approach (Snowdon, Schultz, & Swinburn, 2008), through FGDs with men and women, and key informant interviews with community leaders and clinic nurses. Problem trees provide a systematic approach to understanding diets and identify practical solutions to the problems in a clear and easy to interpret manner (Snowdon, Schultz, & Swinburn, 2008). In total, 10 FGDs and 5 interviews were held, involving more than 35 men and 70 women. During FGDs and interviews, participants completed a series of activities to identify the main foods consumed by households, whether these foods were considered healthy and what that meant for health/nutrition. Key nutrition issues were further explored through the problem tree approach to understand the ‘why’ for each cause to enable the identification of underlying determinants. In each of the Malaita communities, community leaders guided a process to combine separate trees into a single community‐level problem tree (see supporting information Figure S1).

A complementary suite of activities in Western Province were undertaken as part of a separate project aimed at using a biocultural approach to understand place‐based definitions of wellbeing (see detailed methodology in McCarter et al., 2018) and provided an opportunity to compare with the qualitative data collected in Malaita Province. This project used workshops, FGDs and interviews to discuss drivers of change within food systems. Workshops had broad participation among community members and aimed to understand changes over time, aspirations and goals. Based on this, three FGDs with target groups (particularly women and youth) explored in‐depth changes in particular components of the food system (e.g., the role of store‐bought food in disaster situations, or the interactions of different pressures on diets). Key informant interviews (W1, *n* = 15; W2, *n* = 10) further explored trends and issues revealed in the FGDs. We used art to engage with topics in different ways, for example by using visual food diaries to depict actual and ideal meal plates. While we used different qualitative methods in Western and Malaita Provinces, both methods generated community perspectives on nutrition issues and a set of determinants that seek to explain observed trends in diet quality.

### 2.3 Statistical analysis

#### 2.3.1 Wealth indices

To generate wealth indices we used PCA with Varimax rotation (Brown, 2009). Variables included roofing material, flooring material, source of drinking water, source of washing water, number of solar panels, number of mobile phones, and number of radios. Although there was limited variation in wealth among households (see supporting information Table S1), three components explained 66% of the variance. The three components are defined as access to power and communication, access to sanitation, and quality of housing.

#### 2.3.2 Quantitative survey analysis

Statistical analysis of quantitative data was undertaken using SPSS, Version 23, to explore the relationship between women's dietary diversity and education attainment, household wealth, source of household income and nutritional status (BMI, waist to hip ratio). Analyses included backward stepwise regression, Kruskal‐Wallis Test, Spearman Rank correlation, Pearson's correlation and one‐way ANOVA. Differences in average women's dietary diversity, BMI and FSC's among villages was also assessed using one‐way ANOVA and Levene's test.

### 2.4 Ethical considerations

Ethics approval was obtained from the Solomon Islands Ministry of Health and Medical Services (Ethics approval HRE10/16). Participants were included only after they (or their caregiver in case of infants and young children) provided written informed consent. Participation in the study was voluntary and all data were treated confidentially.

## 3 RESULTS

### 3.1 Household characteristics

Participating households between the four study villages were of similar size (mean = 5.6 persons) and age distribution (see supporting information Table S2). The primary sources of income were fisheries and agriculture in three of the four study villages, while the sale of wood and stone carvings provided an important income source at village W1. Households in Malaita relied more on fisheries for income than those in Western Province (see supporting information Table S2). Results of a one‐way ANOVA suggest significant differences between the mean village scores in terms of both access to power and communications (*F*_(3,132)_ = 3.87, *p* = 0.011) and access to sanitation (*F*_(3,132)_ = 36.3, *p* < 0.001). W1 had better access to power and communication assets compared to other villages. M1 and M2 had better access to sanitation than W1 and W2 (see supporting information Table S2).

### 3.2 Nutritional status

Overall, 30.3% of women assessed were overweight and a further 20.7% were obese (Table 1). Based on BMI and waist to hip ratio, almost 64% of women across the study communities had abdominal obesity. BMI and waist to hip ratio were significantly correlated (Pearson's *ρ* = 0.52, *p* < 0.001). There were no significant differences in women's BMI between study communities (ANOVA *F*_(3,110)_ = 3.82, *p* > 0.05) or education attainment (independent sample t‐test *t* = 1.32, *p* > 0.05).

**Table 1:** Nutritional status of women of reproductive age, infants and young children by study community (%)

	Malaita Province		Western Province		Overall
	M1	M2	W1	W2	
**Women of reproductive age (15 to 49 years)**					
*n*	54	32	18	18	122
Underweight	0	0	0	0	0
Normal weight	46.3	56.3	50.0	50.0	50
Overweight	38.9	28.1	38.9	22.2	30.3
Obese	14.8	15.6	11.1	27.8	19.7
Mean waste to hip ratio (SE)	0.86 (0.01)	0.88 (0.01)	0.88 (0.01)	0.86 (0.01)	0.87 (0.006)
Abdominal obesity	64.8	65.6	72.2	50.0	63.9
**Infants and young children (aged 6 to 23 months)**					
n	12	11	6	5	34
Any malnutrition (stunting, wasted or underweight)	25.0	18.2	0	33.3	20.0
Stunted	16.7	18.2	0	20.0	14.7
Wasted	8.3	0	0	0	2.9
Underweight	0	0	0	28.6	4.6
**Infants and young children (aged 2 to 5 years)**					
n	12	16	9	8	45
Any malnutrition (stunting, wasted, or underweight)	50.0	31.2	44.4	37.5	40.0
Stunted	41.6	31.2	22.2	37.5	33.3
Wasted	8.3	0	11.1	0	4.4
Underweight	12.5	5.3	33.3	25.0	15.4

Malnutrition was evident in 20% of infants (aged 6 to 23 months) and 40% of children (aged 2 to 5 years) assessed in this study (Table 1). The most prevalent form of child malnutrition was stunting with 14.7% of infants and young children aged 6 to 23 months and 33.3% of young children aged 2 to 5 years stunted across all sites.

### 3.3 Determinants of malnutrition

#### 3.3.1 Dietary intake

**Figure 1:**
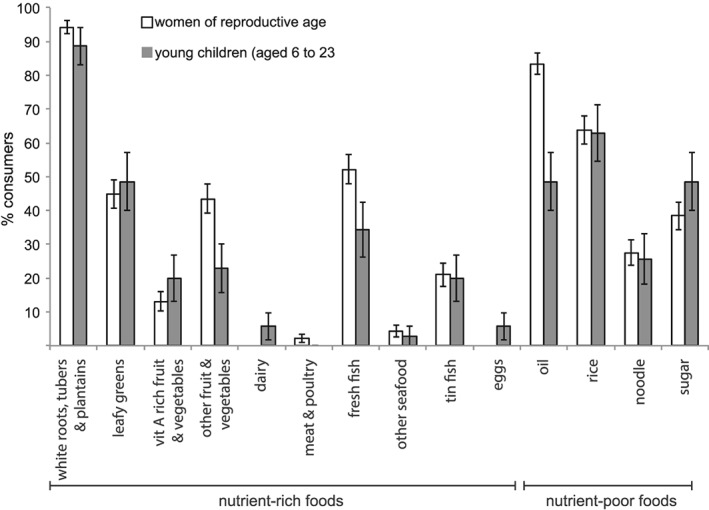
Proportion of women of reproductive age and infant and young children (aged 6 to 23 months) consuming selected nutrient‐rich and nutrient‐poor foods

**Figure 2:**
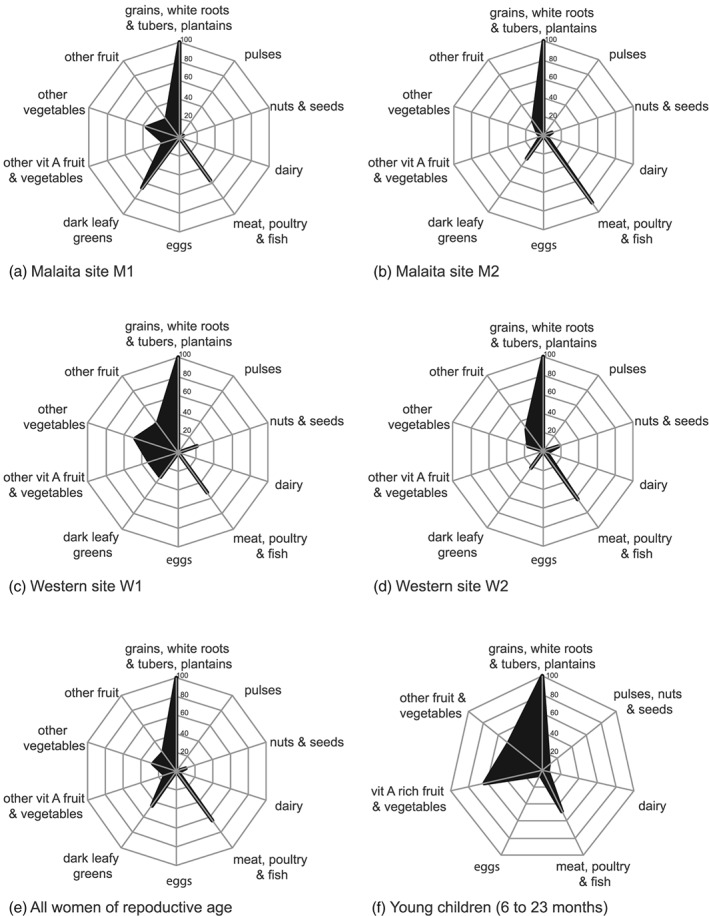
Proportion of women consuming the ten food groups that make up the minimum dietary diversity for women indicator (MDD‐W) at a) site M1, b) site M2, c) site W1, d) site W2, e) women from all sites; and f) proportion of young children (aged 6 to 23 months) from all sites consuming the seven groups that make up the infant and young child feeding dietary diversity score (IYCF‐DD)

All women reported consuming grains, white roots and tubers (mostly sweet potato and rice) and a high proportion (53 – 88% of women) consumed meat, poultry and fish (Figures 1 & 2). Fish was the dominant animal source food; with 37 – 85% of women consuming fresh fish and 15 – 33% consuming canned fish across study sites. A small percentage of women (4%) reported consuming other seafood (principally shellfish) and only 2% consumed other animal source foods (chicken and canned meat). A moderate proportion of women (21 – 66%) consumed dark leafy greens. Less than half of women within each village consumed foods from other food groups: other vitamin A rich fruit and vegetables (0 – 32%), other vegetables (6 – 48%), other fruit (18 – 37%), and nuts and seed (5– 17%). No women reported consuming dairy products, eggs or pulses.

Diets were very low in diversity; only 5.8% of women (pooled across sties) were observed to consume the recommended minimum of five food groups (mean MDD‐W = 2.9 ± 0.09), with most women consuming only 2 or 3 of the 10 food groups that make up the MDD‐W score (Table 2). This would indicate that few women had adequate micronutrient intakes. Backwards stepwise regression of the effect of components of wealth and education attainment suggests that only power and communication (wealth component 1) had an effect on MDD‐W (t = 2.84, *p* = 0.005). There was also no significant difference in MDD‐W between income categories (Chi square = 0.92, *p*>0.05). Additionally, Spearman rank correlation identified no correlation between MDD‐W and BMI. There was however, a significant difference in MDD‐W between villages (ANOVA *F* = 3.06, *p* = 0.03, Levene's *F*_(3, 134)_ = 1.42; *p* = .241). Least Significant Difference post hoc comparison of MDD‐W showed that M1 and W2 were statistically similar, and M2 and W1 were statistically similar which suggests that differences in MDD‐W were not caused by cultural or environmental differences across the provinces.

**Table 2:** Dietary diversity of women of reproductive age and household consumption of sugar and rice across study communities

	Malaita Province		Western Province		Overall
	M1	M2	W1	W2	
**Women's minimum dietary diversity**				
*n*	62	33	19	24	138
% attaining MDD‐W^†^	6.5	3.0	10.5	4.2	5.8
Mean MDD‐W (SE)	3.0 (0.1)	2.6 (0.2)	3.2 (0.3)	2.5 (0.2)	2.9 (0.09)
% consumed 1 food group	3.2	6.1	10.5	16.7	7.2
% consumed 2 food groups	29.0	48.5	21.1	37.5	34.1
% consumed 3 food groups	37.1	30.3	21.1	29.2	31.9
% consumed 4 food groups	24.2	12.1	36.8	12.5	21.0
**Household consumption of selected store foods**				
Sugar					
kg per HH per week	2.5	2.5	1.3	5.7	2.9
g per person per day	59.3	64.8	28.4	110.0	65.3
Rice					
kg per HH per week	5.6	5.1	3.7	3.9	4.9
g per person per day	144.9	131.2	96.3	84.3	122.8

† MDD‐W (minimum dietary diversity of women)

Fewer than a quarter (23%) of infants and young children surveyed had adequately diverse diets (mean IYCF‐DD = 2.8 ± 0.18; Table 3), consuming foods from only 2 or 3 of the 7 food groups. White sweet potato (and/or rice) and vitamin A rich fruits and vegetables (mostly pawpaw and a leafy green) were the dominant foods fed to infants and young children aged 6 to 23 months. Fresh fish was consumed by 34% of infants and young children. Fewer than 9% ate dairy, eggs or pulses/nuts (note the IYCF MDD combines pulses and nuts, only nuts contributed to this food group; Figure 2). Due to a low sample size within each village, comparisons among communities and drivers were not made for infants and young children (Table S3).

**Table 3:** Dietary diversity score, dietary intake and care practice indicators for infant and young children aged 6 to 23 months by study community

	Malaita Province		Western Province		
	M1	M2	W1	W2	ALL
*n*	15	8	4	8	35
Mean IYCF‐DD† score (SE)	2.8 (0.25)	2.25 (0.16)	3 (0.7)	3 (0.5)	2.8 (0.18)
% IYCF‐DD attained	20.0	0.0	25.0	50.0	22.9
% Minimum meal frequency attained	66.7	50.0	75.0	75.0	65.7
% Minimal acceptable diet attained	6.7	0.0	25.0	37.5	14.3
Child ever breastfed (%)	100	100	100	87.5	97.1
Age food introduced (months)	5.0	5.8	7.8	6.4	5.8
Age fish introduced (months)	9.5	9.6	12.0	8.0	9.4

† IYCF‐DD (infant and young child dietary diversity)

#### 3.3.2 Food security and food quality

Borderline or acceptable food consumption scores were obtained by 99% of households across the study communities, indicating most households have adequate food access and availability (see supporting information Table S2). High food consumption scores were driven by the regular consumption of staple foods (root crops and/or rice) and fish. Pulses (a key food group in the food consumption score) were consumed in a small minority of households, however this does not appear to have impacted the ‘food security’ of households, but does highlight the limitations in using such global indicators and the potential for a bias understanding of diets. Across the four study communities, M2 (Malaita) and W2 (Western) were the most ‘food secure’ with more than 90% of households having an acceptable food consumption score. There was a significant difference in food consumption scores between villages (ANOVA *F* = 4.47, *p* = 0.005, Levene's *F*_(3, 134)_ = 1.52; *p* = 0.213). Least Significant Difference post hoc comparison of food consumption scores showed that W1 had a significantly lower mean food consumption scores than the other three villages, suggesting that it is the least ‘food secure’.

In combination with low diversity of diets, a high percentage of women and infants consumed energy‐dense, nutrient‐poor foods, in particular fats and oils, rice and table sugar (Figure 1). Sugar was mostly consumed in ‘tea’, which in rural Solomon Islands villages, often equates to hot water mixed with about 2 tbs (24 g) of sugar. On average, rural households in this study consumed 2.9 (± 0.31) kg sugar week^‐1^ – equal to 65 g pp day^‐1^ (Table 2). Households at W2 consumed the highest amount of table sugar (110 g pp day^‐1^), while households at W1 consumed the least (28 g pp day^‐1^). On average, households consumed 4.9 (± 0.3) kg rice week^‐1^ – equal to 122 g uncooked rice pp day^‐1^ (Table 2). Households in W2 consumed the least rice and the most sugar. Rice consumption was highest at the two Malaita Province sites.

#### 3.3.3 Care resources and practices

IYCF practice indicators showed 97% of infants and young children were ever breastfed (Table 3). Minimum meal frequency was attained by 66% of infants and young children while minimal acceptable diet was attained by only 14%. The mean age for the introduction of semi‐solid foods and fish was 5.8 months and 9.4 months, respectively. The delayed feeding of fish to infants and young children was said to be due to the common belief that fish causes rash and skin issues for infants, concerns with the bones in fish causing choking, as well as cultural beliefs concerning fish and diets.

#### 3.3.4 Health environment and services

Few households in the four communities had access to improved sanitation facilities, with the ocean and/or rivers being the primary location for defecation. In contrast, more households had access to improved drinking water, with the exception of W1, which relied entirely on water from unprotected streams and/or a spring (which was in poor condition because of logging activities). Travel time to the closest available health clinic ranged from a 20‐minute motorized boat ride to a 60‐minute walk.

### 3.4 Community perceptions of nutrition issues: identifying the basic determinants of nutrition

**Figure 3:**
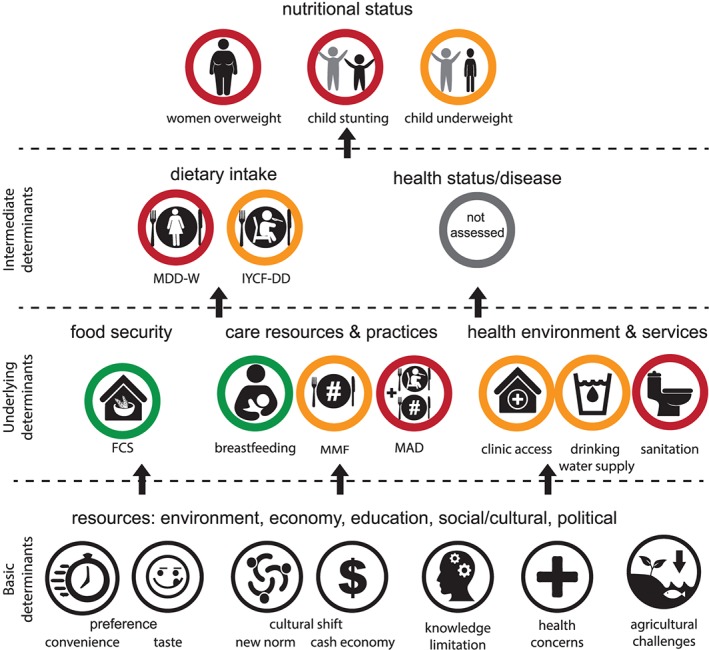
State of key indicators measured in this study (green = good, orange = moderate, red = poor) relative to the UNICEF framework for malnutrition and its immediate, underlying and basic determinants

Communities in Malaita identified high consumption of store foods (e.g. rice, instant noodles and canned tuna) and inadequate consumption of local foods as the main issues impacting on people's health (see supporting information Figure S1). Through the problem tree analysis, several common themes emerged from both sites as the *cause* of these nutrition issues and form some of the basic determinants of malnutrition. These basic determinants included; 1) a preference for imported foods (due to taste and convenience); 2) a shift to a market‐based economy and the associated cultural shift linked to increasing accessibility; 3) lack of basic nutrition knowledge; 4) health concerns (unregulated pesticide use, shellfish contamination from poor sanitation and fears of fish causing child sickness); and 5) decreased agricultural yields associated with declining soil fertility and time burden) (Figures 3 and supporting information Figure S1).

Western Province communities noted similar nutritional issues, including the shift in diets towards store‐bought foods and away from garden produce. Pressures toward changing diets were similar to those noted above. Participants in W1 described how the shift to a cash income has impacted on the nature and focus of garden food production, while both sites identified societal and cultural shifts in the way different foods (e.g., wild leafy greens) were valued. Participants also emphasized environmental variables: at W2, for example, ocean salt spray makes growing fruits and vegetables difficult, while both sites struggled with the impacts of severe weather events (for example, heavy rain followed by intense sun destroyed staple crops at W2, forcing people to purchase store foods).

## 4 DISCUSSION

Our results confirm earlier national studies (SINSO, & SPC, 2009; SINSO, MHMS, & SPC, 2017) in cataloging the prevalence of stunting among infants and young children and overweight and obesity among women in rural communities in the Solomon Islands. Our mixed methods analysis highlights the lack of dietary diversity in these communities, particularly among women and the complexity in the underlying determinants of malnutrition (Figure 3). Though the focal communities cannot be considered representative of rural communities nationally, these issues are likely to be similar in other rural communities in the Solomon Islands.

The nature of the malnutrition challenge in the Pacific region is changing rapidly (e.g. Connell, 2015; Haddad et al., 2015; Horsey et al., 2019; Hughes & Lawrence, 2005; Opio, 1993; and references therein). In this study, the majority of households were “food secure” with regular access to and consumption of fish and staple root crops. The dietary quality of women and young children, however, was poor. Dietary quality of women in the rural Solomon Islands study communities are comparable to women in rural Burkina Faso, West Africa where 5.8% achieved MDD‐W and are in stark contrast to MDD‐W reported for other developing nations, for example, women from rural Bangladesh (22%) and rural Uganda (54.8%) (Martin‐Prével et al., 2015). Similarly, for young children, while initial breastfeeding practices are good, once children reach complementary feeding age, dietary quality deteriorates markedly. The low consumption of nutritious foods and high consumption of low‐quality energy dense foods identified in this study has also been recently reported for an urban centre in Malaita Province (Horsey et al., 2019).

Following HLPE (2017) and Turner et al. (2018) among other authors, we suggest effective responses to these persistent patterns in malnutrition will require interventions at multiple scales within the food environment and broader food system. In response to findings from the present study, below we highlight three domains that offer important opportunities to improve the diets of rural Solomon Islanders. We recognize the multiple risk factors to malnutrition in these rural communities, which relate not only to diets but to other underlying determinants of nutrition outcomes (sanitation, water availability and their external drivers (Haddad, Cameron, & Barnett, 2015)), but confine ourselves here to those factors that directly influence the choices people make about food acquisition and consumption.

### 4.1 Improved agriculture and fisheries

Rural communities in Solomon Islands are facing severe agricultural challenges. Increasing population pressure has resulted in shorter agriculture fallow periods in recent decades, particularly in Malaita (Kabu, 2001; Allen et al., 2006). Subsequent declining soil fertility has impacted productivity and the ability to grow certain foods. For example, several women mentioned that orange‐fleshed sweet potato was previously commonly grown, but there has been a shift away from these varieties due to low yields. Declining fallow periods along with logging activities adjacent to gardens have also resulted in increased garden pest issues (Allen et al., 2006; Minter, Orirana, Boso, & van der Ploeg, 2018).

To date, small‐holder agricultural development in Solomon Islands has focused on high‐value domestic crops (e.g. watermelon, cabbage, tomato, pineapple, peanut and yam); and export commodities, including cocoa and coconut (Bourke et al., 2006; MAL, 2016). To achieve both income generation and nutrition outcomes, nutrition‐sensitive agricultural investments are required to produce and distribute diverse foods with shorter production times and higher nutrition content. Ongoing efforts are also required to address declining soil fertility through improved soil management.

Results from this study highlight an absence of pulses in women and children's diets. While pulses are not a traditional crop in Solomon Islands, agricultural improvement practices have introduced native legumes such as *Mucuna prurient* to improve soil quality (pers comm. Lead farmer, Batolao Farmers Association). Grain legume cropping has been proposed as a potential solution to sustainable food production and human health benefits (Foyer et al., 2016) and could be considered in Solomon Islands.

Fish was an important component of rural diets and offers a range of benefits, including highly bioavailable micronutrients, high quality protein and essential fatty acids (Dignan, Burlingame, Kumar, & Aalbersberg, 2004; Rimm & Mozaffarian, 2006; Michaelsen et al., 2009; Bogard et al., 2015). Whilst fish was widely consumed, there is scope to improve consumption within critical periods, for example within the first 1000 days of life. Efforts must be taken to ensure that fish can continue to play an important role in local diets into the future, in the context of increasing pressures from population growth, climate change and globalization (see Bell et al., 2009).

### 4.2 Improved nutritional knowledge

The overriding weak knowledge of the nutritional value of food was apparent across all communities, which impedes informed choices regarding food consumption. Education and empowerment for individuals, households and communities to make informed food choices is a foundation for improving nutrition (Barker, 2015). While the mode of participatory research used in this study, in itself, was a mechanism to foster empowerment and collective learning (Apgar et al., 2017), there are other opportunities to improve diets. The critical importance of good nutrition in the first 1000 days of life is well‐established in the literature (Victora et al., 2008, Moore, Arefabid, Deery, & West, 2017) but the concept is not well known in Solomon Islands. There is considerable potential to use the concept in policy development and education, because there is a good cultural fit with how Solomon Island households view this formative phase of life (Alcorn, 2010).

Behaviour change interventions have been demonstrated to improve child feeding practices and nutritional status (Bhutta et al., 2013; Caulfield, Huffman, & Piwoz, 1999; Dewey & Adu‐Afarwuah; Shi & Zhang, 2011), however to be effective they need to be implemented through formative research and assessed through impact pathways (Fabrizio, van Liere, & Pelto, 2014). The research conducted as part of this study provides a basis for developing simple impactful messages and provides a framework for developing impact pathways for assessment of change.

Of the ‘top 10’ nutritious leafy green vegetables available locally in rural Solomon Islands villages (Goebel, Taylor, & Lyons, 2014), only three were consumed by women in the 24‐hour period assessed, with slippery cabbage (*Abelmoschus manihot*) the most common leafy green consumed. In rural communities, households commonly grow nutritious leafy greens such as Ofenga (*Polyscias sp)* and Ete (*Pseuderanthemum whartonianum*) as ‘live’ household fences, yet these greens were not part of women and young children's diets. While highly nutritious, such foods may be overlooked as they may be regarded as ‘low status’ foods (Goebel, Taylor, & Lyons, 2014). Further, our study found that in Malaita Province fruit high in beta‐carotene, such as orange‐flesh bananas are available, yet rarely consumed due to their perceived ill‐health effect of ‘making urine orange’. In this regard, understanding social and cultural inhibitions to changing diets and identifying mechanisms to celebrate nutritious local foods will be essential to enhance their consumption. Participants in this study identified the documentation of traditional wild‐harvested foods, as well as cooking demonstrations showcasing simple, fast, flavoursome recipes using locally available foods and advertising healthy foods with simple messaging (e.g. pawpaw are good for healthy eyes) as potential mechanisms for change (see also Foley et al. 2016; Oliver & Berno, 2016).

### 4.3 Reducing consumption of imported goods

Declining fisheries and agriculture productivity have increased time burdens (especially on women) and decreased motivation for household food production. These pressures contribute to preference for more convenient food available in local stores. Store foods (e.g. rice, noodles, tinned tuna, sugar) are now common household foods and are ‘the new norm’ in rural communities. Rice, for example, has a shorter cooking time, a longer shelf‐life, and can feed more people compared with local foods. The general lack of interest in local foods, coupled with a trend toward a market‐based economy, creates the opportunity to buy store foods, which are perceived to have a higher wealth status.

Pacific nations are reliant on imported and processed foods with availability and accessibility driven by economic and political ties, donor aid and trade links (Snowdon & Thow, 2013). Multi‐sector policies have the potential to alter food supply and contribute to healthier populations in the region, yet the impacts of existing policies and policy changes on diets are poorly understood (Thow et al., 2011, Snowdon & Thow, 2013). Sugar sweetened beverage taxes have, for example, been introduced in a number of Pacific countries, yet there remains a paucity of empirical data on the impact of the tax in addressing the NCD crisis (McDonald, 2015). Our findings revealed the high consumption of table sugar (primarily in ‘tea’) and highlights that policy in this sector need to consider the broader food environment and consumption patterns of processed foods. The recent development of a national multi‐sectoral food security, food safety and nutrition policy in Solomon Islands (2016‐2020) provides the basis for collective action to reduce malnutrition in Solomon Islands. However, sufficient capacity and support will be required to enable governments to implement action plans to support this and other nutrition policies and frameworks.

## 5 CONCLUSION

Solomon Islands, and the broader Pacific region face a dire public health and malnutrition situation if current trajectories continue. Progress in nudging those trajectories toward better outcomes will require nutrition‐sensitive interventions aimed at the structural drivers of national food systems and the external food environment (HLPE, 2017; Turner et al., 2018). As important, however, will be to extend the work described here on the local processes that influence how rural Pacific Islanders make choices about the food they acquire and consume (the personal food environment of Turner et al., 2018). Some of those ‘choices’ will be driven by food accessibility and affordability, but education and empowerment will be foundational to enable people to make different and more informed decisions to improve nutrition.

## CONFLICTS OF INTEREST

The authors declare that they have no conflicts of interest.

## CONTRIBUTOR STATEMENT

JA, JB, JMc, SD, JM and NA designed the research; JA, JB, FS, JMc conducted the research; JA, FS and TB analyzed the data; JA, JB, JMc and NA drafted the manuscript. All authors contributed to data interpretation and manuscript revision.

## Supporting information

Data S1. Supporting Information

Table S1 Components of wealth derived from principle component analysis
Table S2 Descriptive household characteristics by study community
Table S3 Descriptive characteristics of women, infants and young children

Figure S1 Nutrition problem tree analysis for Malaita Province site M1 (top) and M2 (bottom).
