# Supplementary material for: Malnutrition in rural Solomon Islands: An analysis of the problem and its drivers
Source: Matern Child Nutr. 2020 Jan 31;16(2):e12921. doi: 10.1111/mcn.12921 (PMC7083421; doi:10.1111/mcn.12921)
Supplement: Supplementary file 2 — Table S1 Components of wealth derived from principle component analysis Table S2 Descriptive household characteristics by study community Table S3 Descriptive characteristics of women, infants and young children [file MCN-16-e12921-s001.docx]

Supporting Information

Table S1 Components of wealth derived from principle component analysis

| **Input variables relating to household wealth** | **Principal Component** | | |
| --- | --- | --- | --- |
|  | **Access to power and communication** | **Access to sanitation** | **Quality of housing** |
| roofing material | 0.214 | -0.104 | **0.753** |
| flooring material | -0.019 | 0.085 | **0.836** |
| source of drinking water | -0.146 | **0.87** | 0.027 |
| source of washing water | 0.096 | **0.89** | -0.042 |
| number of solar panels | **0.751** | -0.084 | 0.181 |
| number of mobile phones | **0.73** | 0.19 | 0.124 |
| number of radios | **0.719** | -0.139 | -0.056 |

Kaiser-Meyer-Olkin Measure of Sampling Adequacy= 0.56; Bartlett's Test of Sphericity Chi-Square = 133.3, *p*<0.001). Bold values reflect strong contribution of input variables to variance explained by components

Table S2 Descriptive household characteristics by study community

|  | **Malaita Province** | | **Western Province** | |
| --- | --- | --- | --- | --- |
|  | **M1** | **M2** | **W1** | **W2** |
| **Household characteristics** | | | | |
| Total number of households in the village | 55 | 36 | 21 | 25 |
| Proportion of households surveyed | 73.3 | 70.6 | 91.3 | 92.6 |
| Mean household size (SD) | 5.8 (0.78) | 5.4 (0.9) | 5.5 (0.56) | 5.6 (0.45) |
| **Household age distribution (%)** |  |  |  |  |
| <2 years | 6.6 | 4.6 | 3.4 | 4.3 |
| 2 to 5 years | 9.7 | 15.4 | 13.8 | 15.1 |
| 6 to 14 years | 26.3 | 30.8 | 25.9 | 30.9 |
| 15 to 49 years | 47.3 | 41.0 | 37.9 | 37.4 |
| >50 years | 10.0 | 8.2 | 19.0 | 12.2 |
| **Primary income source (% of households)** | | | | |
| Fisheries | 21.8 | 47.2 | 4.8 | 0 |
| Agriculture | 63.6 | 36.1 | 28.6 | 64 |
| Livestock | 0 | 2.8 | 0.0 | 0 |
| Paid labour/salary/sell goods | 14.5 | 11.1 | 52.4 | 28 |
| Nothing | 0 | 2.8 | 14.3 | 8 |
| **Household sanitation (% of households)** | | | | |
| Improved | 10.9 | 2.8 | 0 | 8 |
| Non-improved | 89.1 | 97.2 | 100 | 92 |
| **Household drinking water supply (% of households)** | | | | |
| Improved | 76.4 | 52.8 | 0.0 | 44.0 |
| Non-improved | 21.8 | 47.2 | 100.0 | 56.0 |
| Nearest health clinic | 40mins (walk) | 20mins (boat) | 60mins (walk) | 60mins (walk) |
| **Wealth Components (Principle Component)** | | | | |
| PC1 communication & power | 0.17 (0.15) | 0.06 (0.23) | 0.57 (0.25) | 1.3 (0.25) |
| PC2 water supply | 0.89 (0.12) | 0.56 (0.09) | 0.21 (0.05) | 0.88 (0.18) |
| PC3 housing materials | - 0.08 (0.15) | - 0.1 (0.13) | - 0.02 (0.22) | 0.33 (0.18) |
| **Food Consumption Score (% of households)** | | | | |
| poor | 0 | 2.8 | 0 | 0 |
| borderline | 14.5 | 5.6 | 23.8 | 0 |
| acceptable | 85.5 | 91.7 | 76.2 | 100 |

Table S3 Descriptive characteristics of women, infants and young children

|  | **Malaita Province** | | **Western Province** | |
| --- | --- | --- | --- | --- |
|  | **M1** | **M2** | **W1** | **W2** |
| **Women characteristics** |  |  |  |  |
| Mean age (range in years) | 31(15 – 49) | 31 (18 – 49) | 38 (19 – 49) | 37 (23 – 49) |
| Number of women | 62 | 33 | 19 | 24 |
| **Women education level** |  |  |  |  |
| no formal education (%) | 22.6 | 21.2 | 5.3 | 0 |
| any primary (%) | 32.3 | 45.5 | 42.1 | 50.0 |
| any secondary (%) | 35.5 | 27.3 | 26.3 | 37.5 |
| Training college (%) | 3.2 | 0 | 10.5 | 8.3 |
| no response (%) | 6.5 | 6.1 | 15.8 | 4.2 |
| **Infants (6 months to 2 years)** | | | | |
| Number of infants | 15 | 8 | 4 | 8 |
| Mean age (range in months) | 14.1 (6 – 23) | 15.1 (7-23) | 14.7 (8 -23) | 17 (7-22) |
| **Children (2 to 5 years)** | | | | |
| Number of children | 16 | 18 | 7 | 11 |
| Mean age (range in months) | 42.6 (30-56) | 40.2 (25-59) | (44.7 (24 - 59) | 38.4 (26 - 59) |
